# Vision-Based Automated Control of Magnetic Microrobots

**DOI:** 10.3390/mi13020337

**Published:** 2022-02-21

**Authors:** Xiaoqing Tang, Yuke Li, Xiaoming Liu, Dan Liu, Zhuo Chen, Tatsuo Arai

**Affiliations:** 1 Key Laboratory of Biomimetic Robots and Systems, Ministry of Education, State Key Laboratory of Intelligent Control and Decision of Complex System, Beijing Advanced Innovation Center for Intelligent Robots and Systems, and School of Mechatronical Engineering, Beijing Institute of Technology, Beijing 100081, China; tangxiaoqing@bit.edu.cn (X.T.); yuke_li@ruri.waseda.jp (Y.L.); 3120185104@bit.edu.cn (D.L.); 3120215099@bit.edu.cn (Z.C.); tarai118@jcom.zaq.ne.jp (T.A.); 2 Center for Neuroscience and Biomedical Engineering, The University of Electro-Communications, Tokyo 182-8585, Japan

**Keywords:** magnetic microrobots, automated control, path planning, computer vision, obstacle avoidance

## Abstract

Magnetic microrobots are vital tools for targeted therapy, drug delivery, and micromanipulation on cells in the biomedical field. In this paper, we report an automated control and path planning method of magnetic microrobots based on computer vision. Spherical microrobots can be driven in the rotating magnetic field generated by electromagnetic coils. Under microscopic visual navigation, robust target tracking is achieved using PID–based closed–loop control combined with the Kalman filter, and intelligent obstacle avoidance control can be achieved based on the dynamic window algorithm (DWA) implementation strategy. To improve the performance of magnetic microrobots in trajectory tracking and movement in complicated environments, the magnetic microrobot motion in the flow field at different velocities and different distribution obstacles was investigated. The experimental results showed that the vision-based controller had an excellent performance in a complex environment and that magnetic microrobots could be controlled to move to the target position smoothly and accurately. We envision that the proposed method is a promising opportunity for targeted drug delivery in biological research.

## 1. Introduction

The research of microrobots is an essential branch in the field of robotics. Micromanipulations based on microrobots provide a variety of flexible and new methods for many biomedical studies, including functional cellular analysis, disease diagnosis and treatment, targeted drug delivery, and microsurgery monitoring [1,2,3]. There are an increasing number of studies on robots, especially microrobots, in the biomedical field [4,5,6,7,8]. Due to their small size, they cannot be powered by a built–in power supply, so they can only use external drive methods such as piezoelectric [9,10], optical [11], magnetic [12,13], acoustic [14,15,16], and electrical technologies [17]. There are still many aspects that could influence the native state of biological targets, limiting the further application of the existing approaches. For example, microrobots actuated by piezoelectric ceramics require direct contact with biological targets, optical tweezers typically require high laser power to manipulate cells, and electric field-based methods such as dielectrophoresis may affect cell integrity due to Joule heating [17]. Among the approaches, magnetic field driving for microrobots is an auspicious and powerful method. It has attracted considerable attention in many areas, especially in biomedical research due to its low strength, low frequency, strong tissue penetration, biological harmlessness [18,19], and imperviousness to environmental fluid [10] properties.

With the development of control theory, the methods of micro-nano surgery, cellular operation, and targeted delivery are constantly improved and innovated [1,2,3]. Omar Tahri’s team has achieved open/closed–loop, dual–controlled eye surgery using permanent magnets [20]. Octomag, which uses electromagnetic coils, has also been able to use microrobots for targeted delivery of drugs to the eye [21]. However, the reaction time of people is usually more than 0.2 s, and the fatigue caused by the energy consumption of people will make the error rate increase exponentially.

Inflexible operation stimulated the automatic control of the magnetic microrobots, which offers new solutions to areas of precision operations that have traditionally relied on proficiency to reduce errors, especially in complex and multiple–barrier environments. Zhang’s group covered the spore with magnetic nanoparticles and realized the shortest accessible path planning through evolutionary algorithms and particle swarm optimization (PSO)–based path–planning method with fluorescence recognition [22]. Sun’s team also achieved 3D path planning for the robot in a microchannel [23]. Nelson’s group used lattice-based planning in the search-based planning library (SBPL) to realize automatic obstacle avoidance and extraction of protein crystals by robots [24,25,26,27]. Despite these advances, most current control methods are mainly performed by robots with tens or even hundreds of microns in size and precision. With the improvement of microrobot application technology, the smaller scale of microrobots may have greater application potential in the biomedicine field. Xie group realized the complex path planning for automatic targeted delivery of peanut–shaped robots with small dimensions (~3 μm) [13,28]. They also have improved the robot’s flexibility and transport capacity for automated targeted delivery by assembling clusters of robots [12]. However, as the microrobots become smaller and smaller, the complexity and impact of the environment will increase exponentially. Meanwhile, most practical application scenarios are conducted in vivo where the environment is a flowing fluid, while self-correcting control of robot path planning in a fluid environment has not been involved in their studies. Therefore, the control methods of microrobots still have significant limitations in improving the control optimality and accuracy as well as the sensitivity and response speed of environmental perception. In general, the development of automated algorithms for microrobots is still an open and under-explored research topic. Therefore, many basic automation topics still should be addressed, including, but are not limited to, robust tracking of microrobot tools, perception of microrobot environments, and automation of microrobot movements. Additionally, there are already commercial products available in the market to carry out similar tasks efficiently with magnetic controllers, such as MagnebotiX products [29]. However, the complexity of the control system increases the research cost of microrobots and also requires customized design and research for specific tasks.

**Figure 1:**
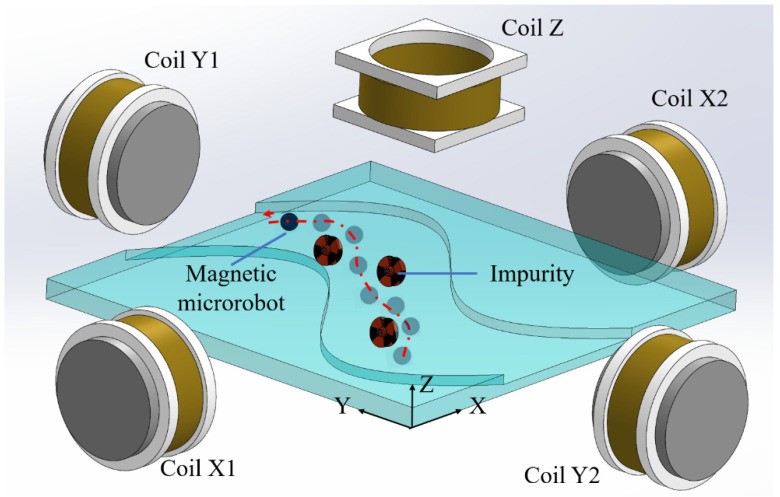
Diagram of a magnetic microrobot moving in the microchannel along the automatic recognition path.

In this article, to address the current control issues, we describe a complete vision-based motion control system for the magnetic microrobot (Figure 1). High resolution in impurities detection can be achieved, even in a cluttered environment containing obstacles such as bubbles or impurities. We demonstrate a strategic and functional approach for detecting and mapping these obstacles in the environment in real-time, planning optimal barrier–free movement routes to arrive at the target position in the shortest time. The magnetic microrobot can perform the trajectory planning in a fluid environment by self-modifying the planned path. The accuracy of the control is basically controlled within 10 microns.

## 2. Materials and Methods

### 2.1. Motion Control of Magnetic Microrobot

**Figure 2:**
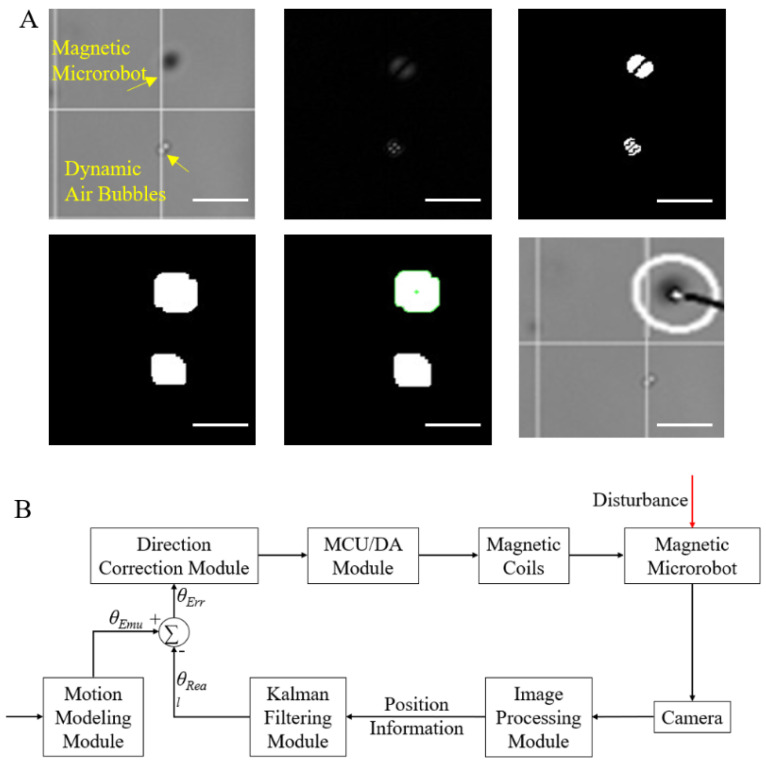
(**A**) Image processing technology, including real images, image difference, binarization, corrosion and expansion images, optimal fitting and contour detection, and reallocation labeling. (**B**) Block diagram of the vision-based closed-loop directional control module. Scale bars: 25 µm.

Motion control of magnetic microrobots includes position detection and prediction with computer vision. Image processing of magnetic microrobots for position detection is conducted as shown in Figure 2A. The detailed process is introduced as follows: the frame difference method is used to make differences between two adjacent pictures to exclude static interference in the background. For dynamic disturbances such as bubbles and impurities, segmentation is performed by binarization, and morphological filtering is performed by corrosion expansion. The experimental results showed that nearly all the impurities in the operating environment could be detected, and the smallest size of impurities is about 1 μm. The microscope can easily make false focusing and double images due to the uneven light reflection on the surface of the magnetic microrobot. Therefore, there will be two overlapping robot pictures after the frame rate has been made different. To address the problem, morphological filtering is used to obtain many discrete points scattered around the actual position of the robot. To improve the accuracy of recognition, we tested several polygon contour fitting and enveloping methods with the OpenCV library (3.2.0) and finally found that the ellipsoidal filtering is better at fitting true centroids from scattered points resulting in more continuous elliptical trajectories. The ellipse center was approximated as the top point of the center of moment drawing of the magnetic microrobot, and the coordinate information was printed and saved. All moment centers were connected to draw the motion trajectory of the magnetic microrobot. Then, an incremental PID (proportion, integral, differential) controller was used to design a directional closed-loop control module to eliminate the errors between the forward direction and the simulation motion direction caused by various resistances or disturbances caused by the internal and external environments of the system (Figure 2B).

In the actual experiment, the frequency of processing feedback information is usually greater than that of simulation information, which leads to the problem that repeated or incorrect two-dimensional state information often appears at some point in the two frames. We used the Kalman filter to predict the two–dimensional state information of the magnetic microrobot in two frames of images and filtered it. At the same time, not all the data information of each control moment was predicted by the Kalman filter. We timed the operation process of the system by setting a timer for the feedback. Only when the time of the image feedback overlapped was the prediction made, and the information was screened according to the results.

### 2.2. Intelligent Obstacle Avoidance Control

**Figure 3:**
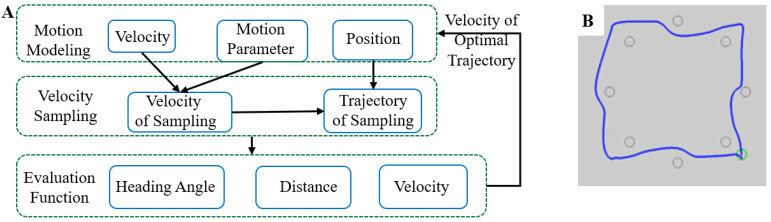
(**A**) Overall framework of DWA. (**B**) The simulation result of obstacle avoidance path planning.

According to the DWA (dynamic window algorithm) implementation strategy proposed in previous research [30], we developed an intelligent motion simulation system to achieve flexible anti-interference control of the magnetic microrobot. The overall framework of the algorithm is shown in Figure 3A.

The trajectory of the magnetic microrobot in a period of time comes from the cumulative sum of displacement increments:

(1) $$x=x+v\Delta t\cdot \cos\left(\theta_{t}\right)$$

(2) $$y=y+v\Delta t\cdot \sin\left(\theta_{t}\right)$$

(3) $$\theta_{t}=\theta_{t}+w\Delta t$$

where *x, y, t,* and $\theta_{t}$ represent the *x*-axis, *y*-axis, time, and the angle between the moving direction and the positive direction of the *x*-axis, respectively. The displacement of the magnetic microrobot is very short in the adjacent Δ*t* moment. The trajectory between two adjacent moments can be regarded as a straight line. Therefore, the movement distance of the robot is v*Δ*t:*

(4) $$\Delta x=v\Delta t\cdot \cos\left(\theta_{t}\right)$$

(5) $$\Delta y=v\Delta t\cdot \sin\left(\theta_{t}\right)$$

When the magnetic microrobot is omnidirectional (having velocity in the axial direction of the robot coordinate system), the variation of the moving distance can be obtained by projecting the moving distance of the magnetic microrobot in the robot coordinate system onto the world coordinate system:

(6) $$\Delta x=v_{y}\Delta t\cdot \cos\left(\theta_{t}+\frac{\pi }{2}\right)=-v_{y}\Delta t\sin\left(\theta_{t}\right)$$

(7) $$\Delta y=v_{y}\Delta t\cdot \sin\left(\theta_{t}+\frac{\pi }{2}\right)=v_{y}\Delta t\cos\left(\theta_{t}\right)$$

The trajectory of velocity can be deduced by summing the axial moving distance superimposed with the displacement increment:

(8) $$x=x+v\Delta t\cdot \cos\left(\theta_{t}\right)-v_{y}\Delta t\sin\left(\theta_{t}\right)$$

(9) $$y=y+v\Delta t\cdot \sin\left(\theta_{t}\right)+v_{y}\Delta t\cos\left(\theta_{t}\right)$$

(10) $$\theta_{t}=\theta_{t}+w\Delta t$$

First, different linear and angular velocities obtained from motion modeling are sampled. The magnetic microrobot is limited by its maximum speed and minimum speed:

(11) $$V_{m}=\left\{v\in \left[v_{min},v_{max}\right],w\in \left[w_{min},w_{max}\right]\right\}$$

Therefore, the actual speed that the magnetic microrobot can achieve in the dynamic window is:

(12) $$V_{d}=\left\{\begin{array}{c} \left(v,w\right)|v\in \left[v_{c}-{\dot{v}}_{b}\Delta t,v_{c}+{\dot{v}}_{a}\Delta t\right]\wedge \\ w\in \left[w_{c}-{\dot{w}}_{b}\Delta t,w_{c}+{\dot{w}}_{a}\Delta t\right] \end{array}\right\}$$

The trajectory can then be calculated by combining different linear and angular velocities. We weighted and normalized smoothing from the three dimensions of azimuth, distance and speed and evaluated each track using the following evaluation function:

(13) G(v,w)=σ(α⋅heading(v,w)+β⋅dist(v,w))+γ⋅velocity (v,w))

Its physical significance is to drive the robot to avoid obstacles and move relatively fast toward the target in the local navigation process. As shown in Figure 3B, in the intelligent obstacle avoidance simulation system, the four vertices of the square were taken as the target points of the movement, and obstacles were set in the movement route. The simulation results showed that the robot successfully avoided all obstacles and accurately reached all target points. Meanwhile, motion control data could also be obtained.

## 3. Experiments and Results

### 3.1. System Setup

**Figure 4:**
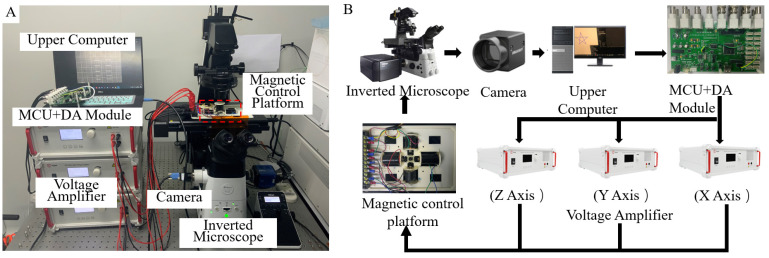
System setup. (**A**) Experimental system of a magnetic microrobot with computer vision. (**B**) System workflow.

The configuration of the magnetic microrobot system is shown in Figure 4. The vision system uses an inverted microscope (Nikon, Eclipse TI2-E, Japan) built on an optical shock-proof platform (SAHT-1510K5, Ming Li, Japan), with the configuration model of a 10× (NA: 0.45) objective lens and black and white industrial camera (Hikvision. MV-CA013-21 μm, 6 fps, China) provide real-time visual image feedback. The magnetic control system is mounted on a three-dimensional control platform under an inverted microscope. Two pairs of electromagnetic coils are symmetrically distributed around the drive space in the X-Y plane, providing a uniform magnetic field to control the direction of motion in the horizontal direction. Another coil is arranged on top of the central operating platform in the Z–axis direction to provide a rotating magnetic field to control the start and stop of movement. The light generated by the *z*-axis light source passes through the central space of the coil, allowing the confocal optical lens of the inverted microscope below to capture the image normally. The control system generates driving signals through the upper computer (2.21-GHz CPU and 16-GB RAM) and the lower computer STM32 MCU + DA (STM32F767ZI + AD5363), which are amplified by three voltage amplifiers in the magnetic control system to drive the electromagnetic coil. A uniform and gradient-free magnetic field is generated in a 5 × 5 mm^2^ workspace. The spherical magnetic microrobots used in this study were ferromagnetic particles (Spherotech) with an about 4 μm diameter (here, we take the size of 4 μm of magnetic beads as an example. Actually, the identical approach can guide nano-scale particles or single or aggregates of micro- and nanoparticles). Ferromagnetic particles are prepared using chromium dioxide coated onto uniform polystyrene particles.

Before the actual experiments, the magnetic bead solution was diluted with pure water. The operation platform is an open chamber made by a 3D printer, and the underside was bonded with glass. We injected the particle solution into the chamber and made sure only a single bead was in the visual field (300 μm × 400 μm) to eliminate the interference between the motions of multiple particles.

### 3.2. Calibration: Magnetic Field System and Robot Motion

The actuation method of the magnetic bead is to exert torque on magnetic beads by a rotating magnetic field with constant and uniform magnetic strength in space. The torque tends to rotate and align the bead moment with the direction of the external field. The torque is related to the net magnetic moment of the magnetic bead (*M*) and the magnetic flux density (*B*), which is denoted as *τ* = *M* × *B*. The rotational actuation on operated particles can be capable of precise control of torque, orientation, and angular velocity of magnetic particles with the applied function signal from three directions.

**Figure 5:**
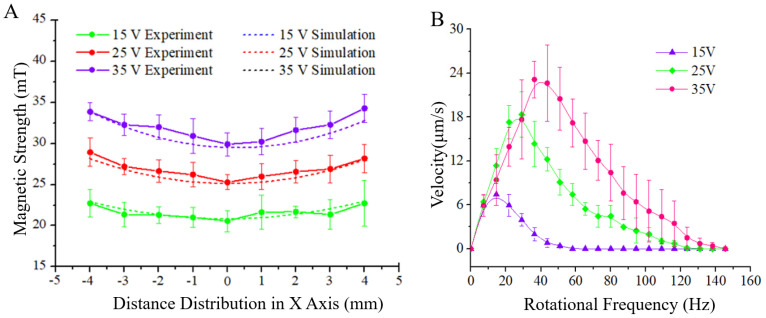
(**A**) Variation in magnetic strength in the workspace under different voltages. (**B**) Relationship between the motion velocity of magnetic microrobots and the frequency of the input signal under various voltages.

Figure 5A shows the simulation results and experimental test results of the magnetic field intensity at different positions within the range of 5 × 5 mm^2^ when the coil voltage is 5 V, 15 V, 25 V, and 35 V. To assess the strength of the magnetic field, high accuracy digital tesla meter was used, which was installed on a three-axis mobile platform driven by a helical micrometer. The mobile platform can change the position of the tesla meter and accurately measure the magnetic field strength at each point in the workspace. The experimental results were in good agreement with the simulation results. It was verified that the magnetic field gradient in the middle part of the magnetic workspace is small in the range of 5 × 5 mm^2^, and the maximum gradient variation is less than 2 mT/mm. It can be considered that a relatively uniform magnetic field suitable for driving the magnetic microrobot was generated. As shown in Figure 5B, the motion of the magnetic microrobot at different voltages and rotation frequencies is calibrated experimentally. The experimental results show that the velocity of the magnetic microrobot is positively correlated with the coil’s input voltage. As the coil’s input voltage increases, so do the magnetic strength and exert torque on magnetic beads. When the magnetic induction intensity is constant, the motion speed of the magnetic microrobot will increase directly with increasing rotation frequency, and reach the peak value when the frequency reaches a certain value. As the rotation frequency increases further beyond the threshold, the motion speed of the magnetic microrobot will gradually decrease and eventually approach zero. Comprehensive analysis shows that the phenomenon is related to the driving principle. The propulsive force to induce the magnetic bead moving forward depends on symmetry breaking in the proximity of the glass boundary and the friction with the glass. Therefore, at a relatively low rotation frequency, the increasing frequency of the rotating magnetic field will lead to an increase in the number of interactions between the magnetic microrobot and the moving contact surface, thus reflecting an increase in the moving speed. When the rotation frequency exceeds the threshold, the increasing interaction number will decrease the interaction time. At the same time, the slippage probability of the magnetic microrobot would increase continuously, so the velocity of beads would decrease after a certain maximum threshold frequency of the field. In this respect, the employing of vision–based automated control of magnetic microrobots could help choose maximum propulsion velocity by comparing the moving velocity under the different frequencies of rotating magnetic fields. The results also reflect that the peak velocity increases with increasing magnetic induction intensity at the threshold frequency. Within the allowable voltage range in our experimental system, the motion speed is maximal when the rotation frequency and voltage of the input signal are 45 Hz and 35 V, respectively. Additionally, compared to the publications [31,32,33], our magnetic achiral bead achieved propulsion depending on symmetry breaking in the proximity of the glass boundary instead of moving in viscous environments and far from surfaces. Therefore, the motion properties could have some differences.

### 3.3. Vision-Based Navigated Locomotion

**Figure 6:**
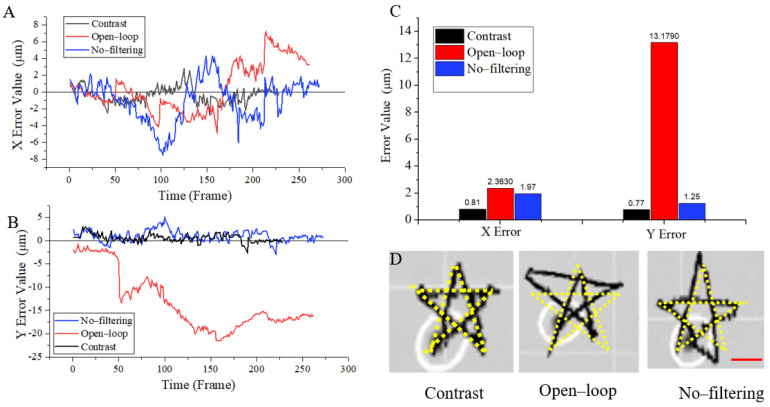
(**A**–**C**) The error in the X-axis and Y-axis directions for contrast, open–loop, and no–filtering systems. The contrast system is conducted with Kalman filtering and closed-loop control. (**A**) Error variation trend in the X direction. (**B**) Error variation trend in the Y direction. (**C**) The average value of the absolute error in X and Y direction. (**D**) Superposition diagram of the simulated path and actual moving path, in contrast, open–loop and no–filtering systems. Scale bars: 30 µm.

To verify the performance of the closed-loop system for the magnetic microrobot, and eliminate the chance of different routes on the control results, we control the magnetic microrobot to move along a pentagram path due to its properties of symmetry and complexity, which contains five directions of motion. Therefore, it is more intuitive to observe the control results using the pentagram path for navigation trajectory. The statistical results are presented in Figure 6. We observed and analyzed the trajectory and errors of magnetic microrobots. Experimental results of the single–function control system were compared with those of the comprehensive control system by making every subfunction of the system a single variable.

As seen from Figure 6C, when the system built in this paper is used to control robot movement in low-speed fluid, the average absolute error in the X direction is about 0.81 μm. The absolute value of the mean error in the Y direction is about 0.77 μm. They are about one-fifth in body length. At the same time, we can see that the overall error fluctuations are very small; it is basically less than 2 μm. In the other two systems, the error of the open–loop system in the X and Y axial directions are about half the body length and four times the body length, respectively. The error is about half of the body length in the system without the Kalman filter. The system proposed in this paper compares the open–loop system; the absolute value of the mean error in the X direction decreased by about 1.55 μm. The absolute value of the mean error in the Y direction decreases by about 12.41 μm. Compared with the system without the Kalman filter, the absolute value of the mean error in the X direction decreases by about 1.16 μm. The absolute value of the mean error in the Y direction decreased by about 0.48 μm. At the same time, we can also see that the control of the closed–loop system is much more stable than that of the open–loop system and the system without the Kalman filter in the whole moving process. By analyzing the error variation trend in Figure 6A,B and combining it with the movement route in Figure 6D (Videos S1–S3), we found that the errors of the open–loop system and the system without the Kalman filter increased proportionally with time, and accumulated when moving in the same direction. The closed–loop system also corrects errors anytime and anywhere to improve the stability and accuracy of magnetic control.

**Figure 7:**
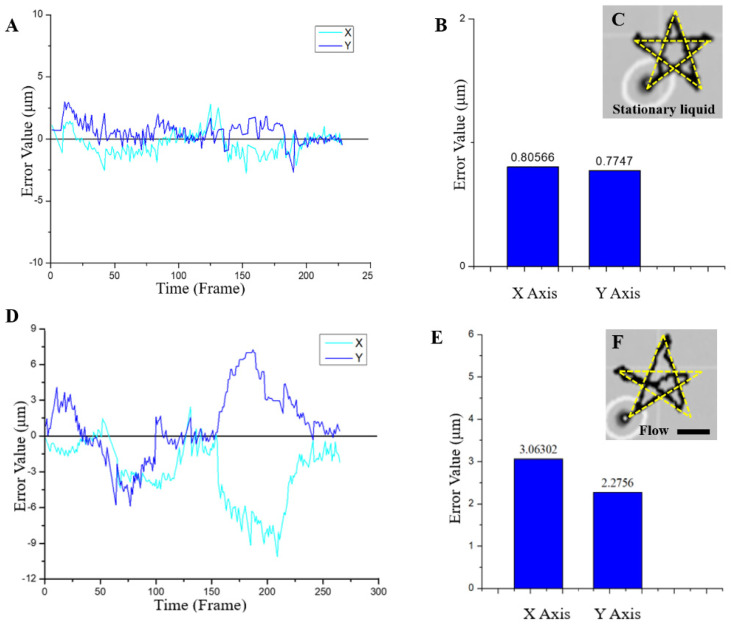
Movement performance of the magnetic microrobot working in static and flowing liquids. Closed-loop control and the Kalman filter have been integrated into the system. (**A**–**C**) The error in the direction of the *X*–axis and *Y*–axis in the static (low velocity) flow. (**A**) Error variation trend. (**B**) The average value of the absolute error. (**C**) Superposition diagram of the simulated path and actual moving path. (**D**–**F**) The error in the direction of the *X*–axis and *Y*–axis in the high–velocity flow (12 μm/s). (**D**) Error variation trend. (**E**) The average value of the absolute error. (**F**) Superposition diagram of the simulated path and actual moving path. Scale bars:10 µm.

We integrate closed-loop control and the Kalman filter into a single system and placed the moving magnetic robot in static and flowing liquid (1 to 12 μm/s) to observe and analyze the motion trajectory and error of the magnetic microrobot. As shown in Figure 7A,B, the magnetic robot moved along the simulated path in static liquid with a relatively small error, and the average values of the absolute errors of X and Y were all less than 1 μm. The actual moving trajectory of the robot in the experiments is also shown in Figure 7C (Video S3). When the magnetic microrobot was driven to move along the pattern path in a flowing liquid, the absolute value of the average error in the X direction was 3.06 μm, the absolute value of the mean error in the Y direction was 2.28 μm (Figure 7D,E). Both are about three-quarters in body length. The actual moving trajectory of the robot in the experiments is also shown in Figure 7F (Video S4). These errors were smaller than those generated by the magnetic microrobot motion under control. The experimental results indicated the validity of our proposed control method.

**Figure 8:**
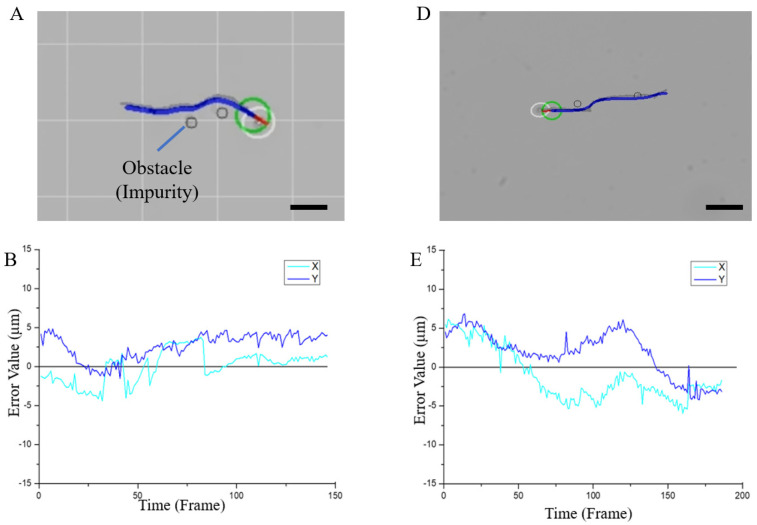

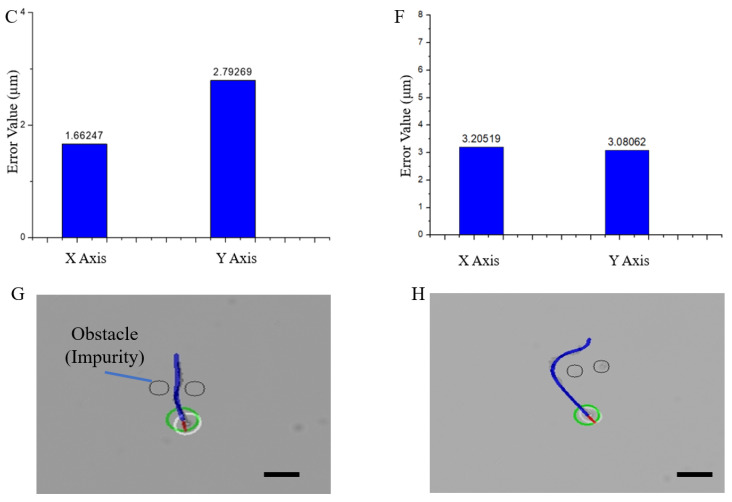
Experimental results of obstacle avoidance and trajectory planning in two situations. (**A**–**C**) Multiple adjacent obstacles in the motion space. (**A**) Superposition diagram of the simulated path and actual moving path. (**B**) Error variation trend. (**C**) The average value of the absolute error in the X and Y directions. (**D**–**F**) Multiple obstacles at large distances in the motion space. (**D**) Superposition diagram of the simulated path and actual moving path. (**E**) Error variation trend. (**F**) The average value of the absolute error in the X and Y directions. (**G**,**H**) Intelligent path planning motion control in the field with obstacles at different spacings. The motion trajectory drawn by the motion simulation system and the actual motion trajectory of the magnetic microrobot is superimposed. Scale bars are 30 µm.

To verify the feasibility of the path planning algorithm and the performance of the obstacle avoidance simulation system, we conducted experiments in two situations with intelligent obstacle avoidance technology. In the first situation, the magnetic microrobot was controlled in the field with adjacent obstacles (Figure 8A and Video S5). We can observe that the robot can continuously pass two adjacent obstacles. In the second situation, the magnetic microrobot was controlled in the field with obstacles at large distances (Figure 8D and Video S6). We can see that the robot can move in a straight line after avoiding the first obstacle and then cross over the second obstacle successfully to find the shortest path. The errors of these two motions in the X and Y directions are shown in Figure 8C,F. The errors of two different intelligent obstacle avoidance movements in different directions were within 4 μm. The fast–intelligent obstacle avoidance movement was realized while matching the navigation path. The selected target point was reached by automatically avoiding obstacles as quickly as possible. At the same time, we verified the intelligence of the simulation system by changing the distance between obstacles. The statistical results of the errors shown in Figure 8C,F also proved that the errors in the whole motion control process are very small, and the path navigation is very accurate. Additionally, according to the experiments, the robot was controlled to pass through obstacles in a straight path to the target point when the distance between obstacles was proper, while the robot was driven to take a detour and reselect the shortest possible path to reach the target point when the distance between obstacles was too small to pass. The critical distance between obstacles is about 10 μm, and the typical experimental results are shown in Figure 8G and Video S7 and Figure 8H and Video S8, respectively.

## 4. Conclusions

This paper proposes a closed-loop motion control method for magnetic microrobots in the range of a few microns based on computer vision. High resolution in target detection can be achieved in a cluttered environment containing obstacles such as bubbles or impurities. The strategic and functional approach was demonstrated for detecting the obstacles in the environment in real-time and planning optimal barrier-free movement routes to arrive at the target position in the shortest time. Additionally, the automation control of magnetic microrobot could perform the trajectory planning in a flowing fluid environment by self–modifying the planned path with a relatively high resolution, which lays the foundation for research on the environment perception of robots. Experiments verify the ability and robustness of the magnetic controller to independently plan and modify the movement path and avoid random obstacles.

Additionally, our research has demonstrated good properties in vitro using automated magnetic control with a vision system and showed potential application prospects in vivo for targeted therapy, drug delivery, and so on. To address the interference induced by non-transparent tissue–based complex environments, more advanced methods, such as fluorescent labeling or magnetic resonance imaging, could be used to distinguish and control the microrobot from the environment based on the characteristics of the enhanced contrast in the future.

## Data Availability

Data are contained within the article or Supplementary Materials.

## Supplementary Materials

The following are available online at https://www.mdpi.com/article/10.3390/mi13020337/s1, Video S1: The magnetic microrobot moving in the open–loop system, Video S2: The magnetic microrobot moving in the system without the Kalman filter, Video S3: The magnetic microrobot moving in contrast system, Video S4: The magnetic microrobot moving in the flowing fluid, Video S5: The magnetic microrobot moving in the field with adjacent obstacles, Video S6: The magnetic microrobot moving in the field with obstacles at large distances, Video S7: The magnetic microrobot passing through obstacles, Video S8: The magnetic microrobot passing around obstacles.
